# An old medicine as a new drug to prevent mitochondrial complex I from producing oxygen radicals

**DOI:** 10.1371/journal.pone.0216385

**Published:** 2019-05-02

**Authors:** Dominique Detaille, Philippe Pasdois, Audrey Sémont, Pierre Dos Santos, Philippe Diolez

**Affiliations:** 1 IHU Liryc, L’institut de rythmologie et modélisation cardiaque, Fondation Bordeaux Université, Pessac-Bordeaux, France; 2 Université de Bordeaux, Bordeaux, France; 3 INSERM, Centre de recherche Cardio-Thoracique de Bordeaux, Bordeaux, France; 4 Centre Hospitalo-Universitaire de Bordeaux (CHU), Pôle Cardio-thoracique, Pessac, France; Virginia Commonwealth University, UNITED STATES

## Abstract

**Findings:**

Here, we demonstrate that OP2113 (5-(4-Methoxyphenyl)-3H-1,2-dithiole-3-thione, CAS 532-11-6), synthesized and used as a drug since 1696, does not act as an unspecific antioxidant molecule (i.e., as a radical scavenger) but unexpectedly decreases mitochondrial reactive oxygen species (ROS/H_2_O_2_) production by acting as a specific inhibitor of ROS production at the I_Q_ site of complex I of the mitochondrial respiratory chain. Studies performed on isolated rat heart mitochondria also showed that OP2113 does not affect oxidative phosphorylation driven by complex I or complex II substrates. We assessed the effect of OP2113 on an infarct model of *ex vivo* rat heart in which mitochondrial ROS production is highly involved and showed that OP2113 protects heart tissue as well as the recovery of heart contractile activity.

**Conclusion / Significance:**

This work represents the first demonstration of a drug authorized for use in humans that can prevent mitochondria from producing ROS/H_2_O_2_. OP2113 therefore appears to be a member of the new class of mitochondrial ROS blockers (S1QELs) and could protect mitochondrial function in numerous diseases in which ROS-induced mitochondrial dysfunction occurs. These applications include but are not limited to aging, Parkinson’s and Alzheimer's diseases, cardiac atrial fibrillation, and ischemia-reperfusion injury.

## Introduction

The free radical theory of aging suggests that free radical-induced damage to cellular structures is a crucial event in aging [1]; however, clinical trials on antioxidant supplementation in various populations have not successfully demonstrated an anti-aging effect [2]. Current explanations include the lack of selectivity of available antioxidants for the various sources of oxygen radicals and the poor distribution of antioxidants to mitochondria, which are now believed to be both the primary sources of reactive oxygen species (ROS) and primary targets of ROS-induced damage [3]. Indeed, mitochondrial dysfunction that occurs due to accumulation of oxidative damage [4] is implicated in the pathogenesis of virtually all human age-related diseases [5, 6], including cardiovascular and neurodegenerative diseases, cancer, and diabetes [7–12], as well as ischemia-reperfusion injury [13].

Given the key role of age-dependent mitochondrial deterioration in aging [4], there is currently a great interest in approaches to protect mitochondria from ROS-mediated damage. Mitochondria are not only a major source of ROS but also particularly susceptible to oxidative damage. Consequently, mitochondria accumulate oxidative damage with age that contribute to mitochondrial dysfunction [4]. Cells and even organelles possess several protection pathways against this ROS-mediated damage given that local protection is fundamental to circumvent the high reactivity of ROS. Therefore, mitochondria appear as the main victims of their own ROS production, and evidence suggests that the best mitochondrial protection will be obtained from inside mitochondria. This conclusion has driven several potential therapeutic strategies to improve mitochondrial function in aging and pathologies. Antioxidants designed for accumulation by mitochondria *in vivo* have been developed [2, 14] and are currently being thoroughly tested for mitochondrial protection [15–17]. Given that functional mitochondria are characterized by a very high proton gradient, mainly represented by a negative-internal membrane potential gradient [18], lipophilic cationic compounds accumulate inside the mitochondrial matrix as they may cross the lipid bilayer barrier given the electrical gradient. Therefore, mitochondria-targeted antioxidants are essentially cationic lipophilic drugs combined with a quinone moiety with radical scavenging properties. The growing interest in ROS production associated with diseases has elicited numerous clinical trials that have also demonstrated that uncontained ROS reduction in cells is deleterious, and it appears that an adequate balance of ROS production is necessary for correct cell function [2]. As a consequence, there is also a growing interest in the selective inhibition of ROS production of mitochondrial origin that would not affect cellular signalization involving either mitochondrial [19] or cytosolic ROS production [20, 21]. Conditions of high ROS production in mitochondria are now better characterized [7, 22–24], and it appears that ROS may be produced at multiple sites of the respiratory chain in mitochondria. Maximal ROS production occurs under conditions of high reduction of electron transporters, chiefly quinones, and high membrane potential values. Paradoxically, these conditions are satisfied when mitochondrial oxidative phosphorylation is low (low cellular ATP turnover) [25, 26] or under low oxygen conditions (hypoxia, inhibition of terminal oxidase) [13].

The molecule OP2113 (Anetholtrithion, or 5-(4-methoxyphenyl)dithiole-3-thione—CAS number 532-11-6) has been marketed in many countries and used in human therapy in certain countries including France, Germany, and China for its choleretic and sialogogic properties. Anetholtrithion also exhibits chemoprotective effects against cancer and various kinds of toxicity caused by some drugs and xenobiotics [27]. These chemoprotective effects appear to be mainly due to its antioxidant properties [28–30]. The most typical indications for which anetholtrithion is currently used include increasing salivary secretion in patients experiencing dry mouth. It is also indicated as an adjunctive therapy for cholecystitis, gallstone, indigestion, and acute/chronic hepatitis (see DrugBank database [31]). Anetholtrithion and derivatives have also been tested for their properties as H_2_S donors and therapeutic effects [27, 32–34].

However, until now, no precise mechanism of action has been described for this molecule. Considering the high lipophilicity of OP2113, which represents a promising characteristic for mitochondrial targeting, we investigated the effect of OP2113 on mitochondrial ROS/H_2_O_2_ production. Here we show that OP2113 decreases ROS/H_2_O_2_ production by isolated rat heart mitochondria. Interestingly, it does not act as an unspecific antioxidant molecule (*i*.*e*. as a radical scavenger), but as a direct specific inhibitor of ROS production at site I_Q_ of complex I of the mitochondrial respiratory chain, without impairing electron transfer.

## Results

### OP2113 does not inhibit mitochondrial oxidative phosphorylation

**Fig 1:**
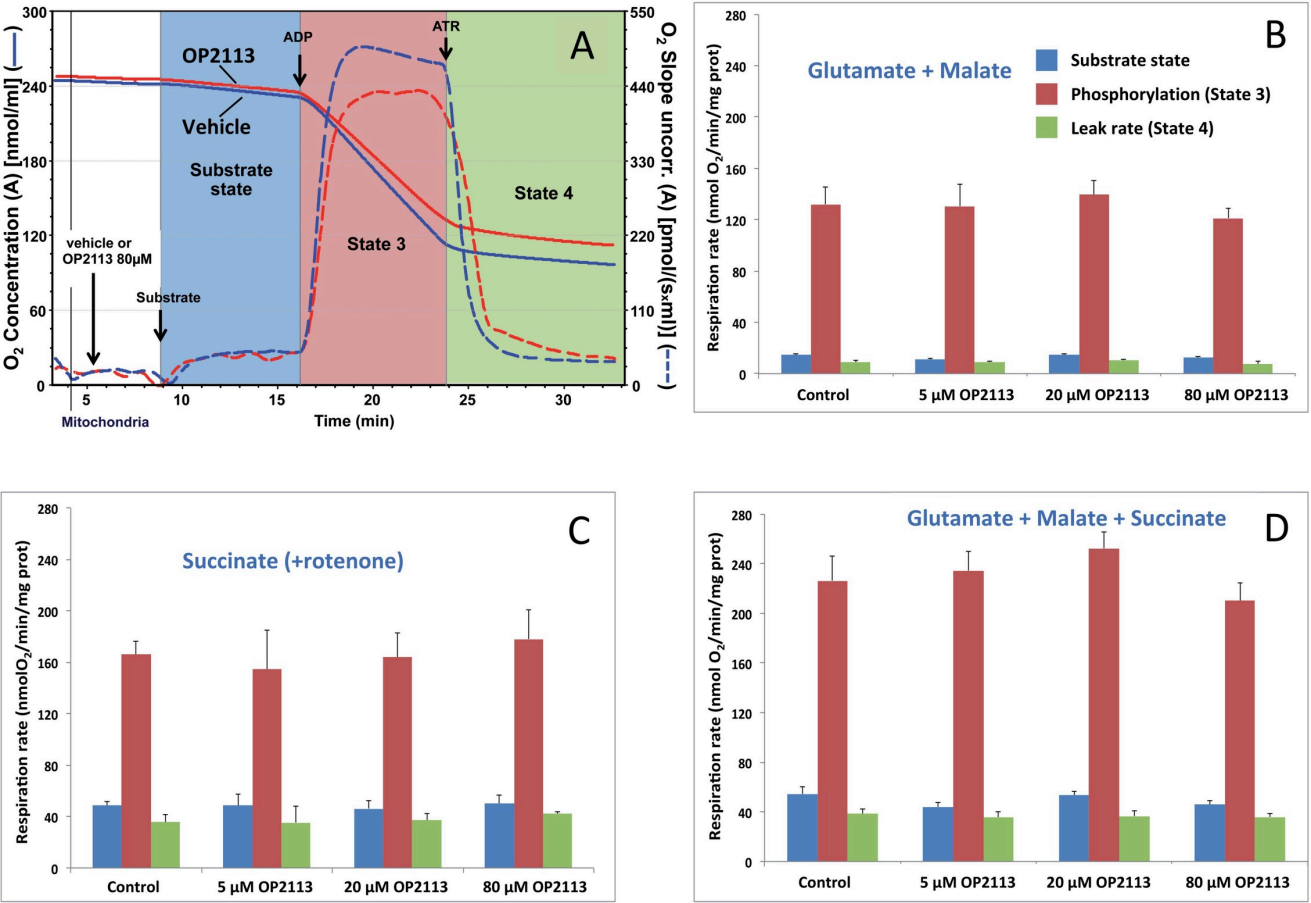
The lack of an effect of OP2113 on mitochondrial oxidative phosphorylation. **Panel A**: This diagram illustrates a classical respiration assay and presents O_2_ concentration (solid lines) and O_2_ consumption slope (dotted lines) of rat heart mitochondria after a short incubation in the presence of the vehicle (blue lines) or 80 μM OP2113 (red lines). Respiratory substrates (glutamate + malate in this assay) were added, triggering the onset of oxygen consumption (substrate state, blue background) and then phosphorylation was promoted by the addition of 1 mM ADP in order to obtain the maximal oxidative phosphorylation rate (State 3, red background) [4, 35]. Finally, atractyloside (ATR), which inhibits the ADP/ATP translocator, was added to yield the mitochondrial leak rate (green background) under non phosphorylating conditions (State 4). **Panels B to D**: The three energetic states were studied in the presence of increasing concentrations of OP2113 (5, 20, and 80 μM), and with mitochondria oxidizing different respiratory substrate combinations: glutamate + malate (**B**), which feeds electrons to complex I; succinate in the presence of rotenone (**C**), supplying electrons to complex II; and glutamate + malate + succinate (**D**) feeding electrons to both complexes I & II. Results are based on 3 independent experiments. No significant differences in mitochondrial respiration rates were noted after the addition of OP2113.

Using the classical oxygraph method, we first verified directly that the OP2113 compound did not affect oxidative phosphorylation or mitochondrial integrity using mitochondria isolated from rat heart. The substrate combination that fed electrons to the entire respiratory chain (see legend to Fig 1) was chosen given that it most closely resembles *in vivo* conditions where metabolism and Krebs cycle are active inside mitochondria and both NADH (complex I) and succinate (complex II) are oxidized by the respiratory chain. No statistically significant differences were observed regarding the presence of OP2113 for the large range of concentrations tested here (Fig 1), demonstrating that OP2113 has no effect on mitochondrial oxidative phosphorylation, respiratory chain activity, ATP synthesis, or mitochondrial inner membrane integrity (leak rate, in green) under these conditions. Overall, these results confirm the absence of any harmful effect of OP2113 on mitochondrial energetics under these conditions.

Since it was not possible to measure more precisely the effect of OP2113 on the specific activity of complex I due to spectrophotometric interferences, we have been advised to assay by polarography the effect of OP2113 on rotenone-sensitive NADH oxidase activity by broken (frozen-thawed) mitochondria (S1 File), assimilated to complex I [36]. Interestingly, this activity turned out to be almost 30 times higher than the activity of oxidative phosphorylation driven by complex I substrates presented in Fig 1 (respectively 3500 versus 130 nmol O_2_/min/mg prot). Due to this huge NADH oxidase activity as compared to complex I oxidative phosphorylation, we had to use a much lower protein content in the assay, while keeping the same drug to protein ratio. We could detect an inhibition (about 8%) of NADH oxidase activity on broken mitochondria starting at the equivalent of 20 μM (200 nmol OP2113 / mg of mitochondrial protein) and increasing to 45% for 80 μM (800 nmol OP2113 / mg of mitochondrial protein). However, due to the difference in activity, even at the higher OP2113 concentration the activity of rotenone-sensitive NADH oxidase is still 15 times higher than complex I driven oxidative phosphorylation rate. These results explain the total absence of effect of OP2113, even at very high concentration (80 μM), on complex I-driven oxidative phosphorylation by intact heart mitochondria.

### OP2113 specifically inhibits mitochondrial superoxide/H_2_O_2_ production

**Fig 2:**
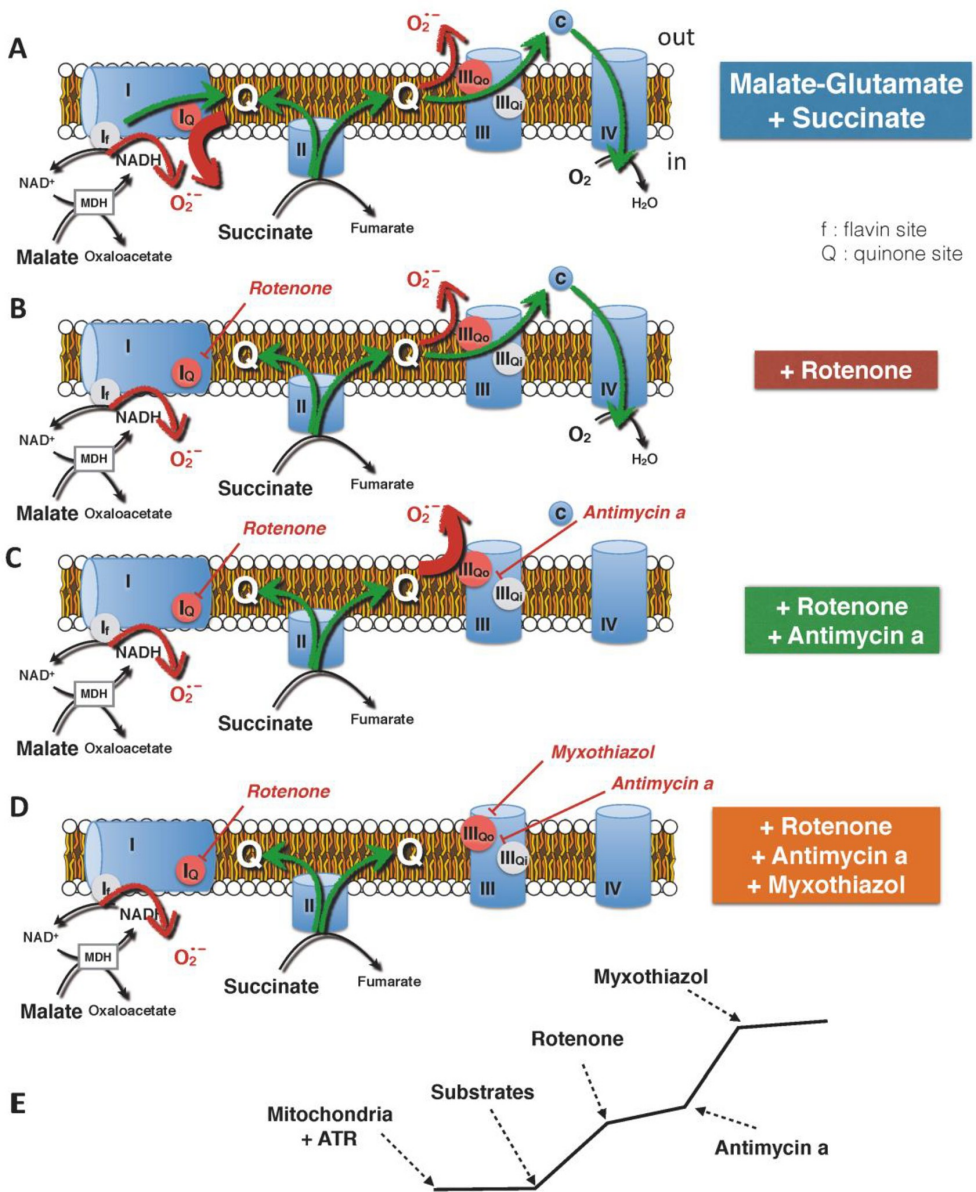
Main sites of oxygen radical production by isolated mitochondria. This scheme gives background information regarding the potential sites for ROS production, using different mitochondrial-targeted drugs in a protocol specifically designed for a study of the effect of a compound on mitochondrial ROS/H_2_O_2_ production. When mitochondria are energized by a combination of complex I (malate-glutamate) and complex II (succinate) substrates and in the absence of specific inhibitors of the complexes, ROS production is considered as mainly derived from reverse electron transport (RET) at site I_Q_ (A). Of note, ROS produced by complex I, either at site I_Q_ (quinone site) or at site I_f_ (flavin site), are delivered to the inner- (matrix-) side of the inner mitochondrial membrane. In the presence of rotenone, a specific inhibitor of complex I which blocks RET, ROS production is thought to occur predominantly at site III_QO_, possibly with residual production at site I_f_ (B) [24]. If complex III is inhibited as it is the case in the presence of antimycin a, the reduced to oxidized quinone ratio increases due to complex II activity and triggers an increase in ROS production, essentially at site III_QO_ (C). Finally, myxothiazol (inhibitor of complex III site III_QO_) is supposed to block complex III ROS production, and the remaining production is usually ascribed to the flavin site of complex I for which there is no known inhibitor (D)[25]. However, due to matrix antioxidant machinery, the possibility that some ROS/H_2_O_2_ produced in the matrix may escape to the measurement has been suggested from experiments carried out with submitochondrial particles [37]. A typical recording of ROS production kinetics by mitochondria during the designed inhibitor sequence is presented in E.

We further tested the effects of OP2113 on ROS production by mitochondria under various conditions. As previously stated, mitochondrial ROS production is highly dependent on mitochondrial bioenergetic state, and maximal production occurs under conditions of high reduction of electron transporters and high membrane potential. These conditions are fulfilled in the presence of ATR (inhibition of ATP/ADP translocator; green bars, Fig 1B to 1D). Under these conditions ROS are produced at different sites of the respiratory chain [7, 24]. The main sites of production are located at complex I and III, where large changes in potential energy of electrons occur [5, 22, 25]. These complexes also allow proton pumping [18]. Based on the work of M.D Brand and colleagues [21, 24, 25], we designed a series of inhibitor titrations to decipher OP2113 action on ROS production by the mitochondrial respiratory chain under conditions of maximal ROS production. The schematic representation of these experiments and of their rationale is presented in Fig 2.

**Fig 3:**
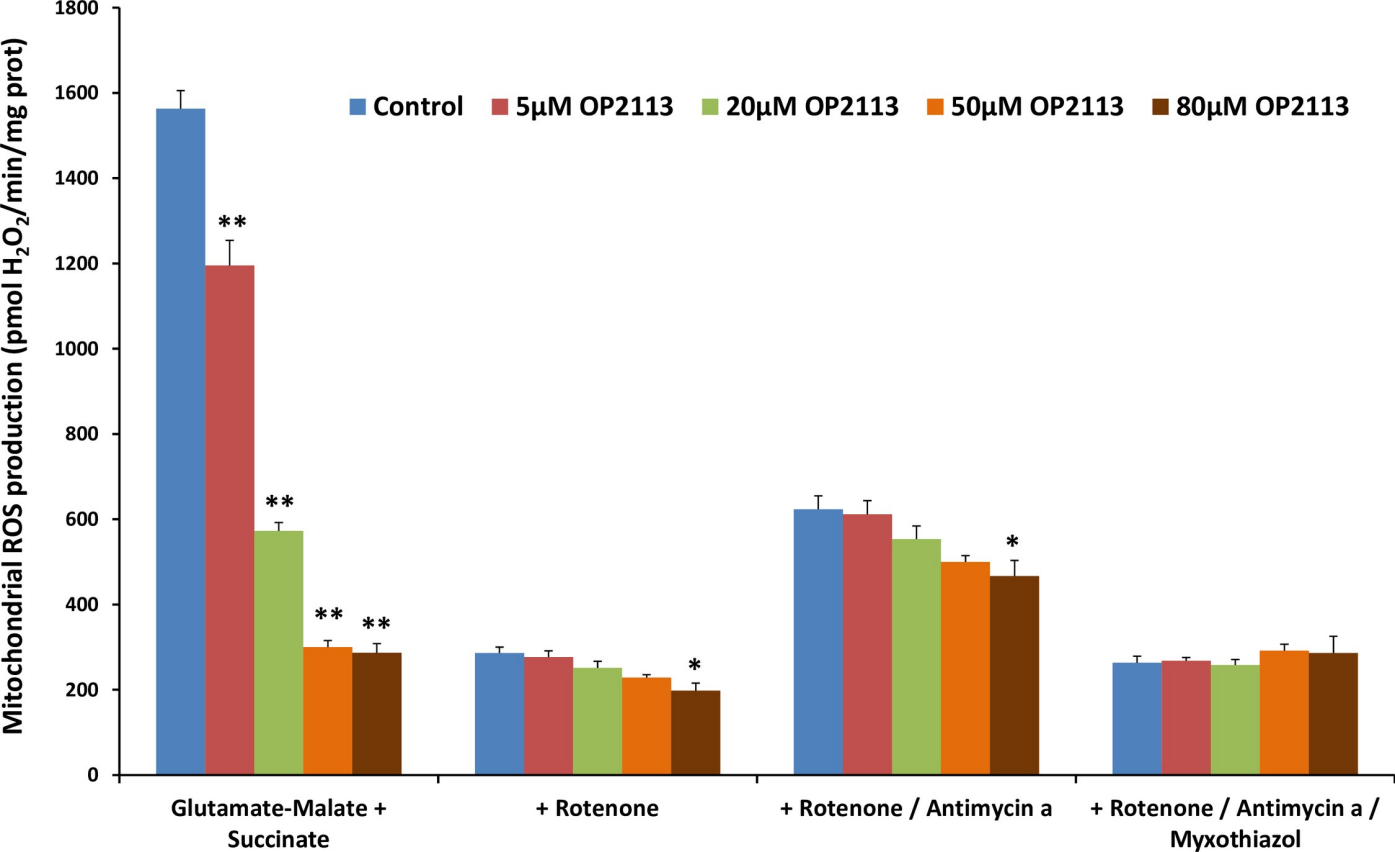
Effect of OP2113 on ROS/H_2_O_2_ production by isolated rat heart mitochondria. The rate of ROS/H_2_O_2_ production by isolated rat heart mitochondria respiring on glutamate + malate + succinate was measured under the different conditions described in Fig 2 in the presence of increasing concentrations of OP2113 (5 to 80 μM). In the absence of specific inhibitors of the complexes (see Fig 2A), ROS/H_2_O_2_ production is maximal and is mainly derived from reverse electron transport at site I_Q_ (see comments in Fig 2). Following blockade of reverse electron transport by addition of rotenone (1.5 μM) (Fig 2B), ROS/H_2_O_2_ production is reduced and occurs essentially at site III_QO_. The subsequent addition of Antimycin a (2 μM) (Fig 2C), which blocks the transfer of electrons to oxygen, increases this ROS/H_2_O_2_ production. Finally, myxothiazol (0.2 μM) blocks ROS/H_2_O_2_ production at site III_QO_ (Fig 2D). Data are based on 4 independent experiments, each performed in duplicate. *P < 0.05, **P < 0.005 *versus* control group.

Fig 3 presents the effect of increasing OP2113 concentrations (from 5 to 80 μM) on ROS/H_2_O_2_ production measured under the different conditions depicted in Fig 2. Results chiefly show that once rotenone has been added to the assay, ROS/H_2_O_2_ production becomes insensitive to OP2113 even at high concentrations, irrespective of the site involved. Thus, the only condition under which OP2113 is active in our assay is the condition where ROS are produced at the level of complex I (site I_Q_ sensitive to rotenone). These results demonstrate that OP2113 significantly decreases ROS/H_2_O_2_ production (by approximately 80%) under conditions where complex I is the main producer (before addition of rotenone), while no significant effect was observed under all other conditions (after addition of rotenone).

**Fig 4:**
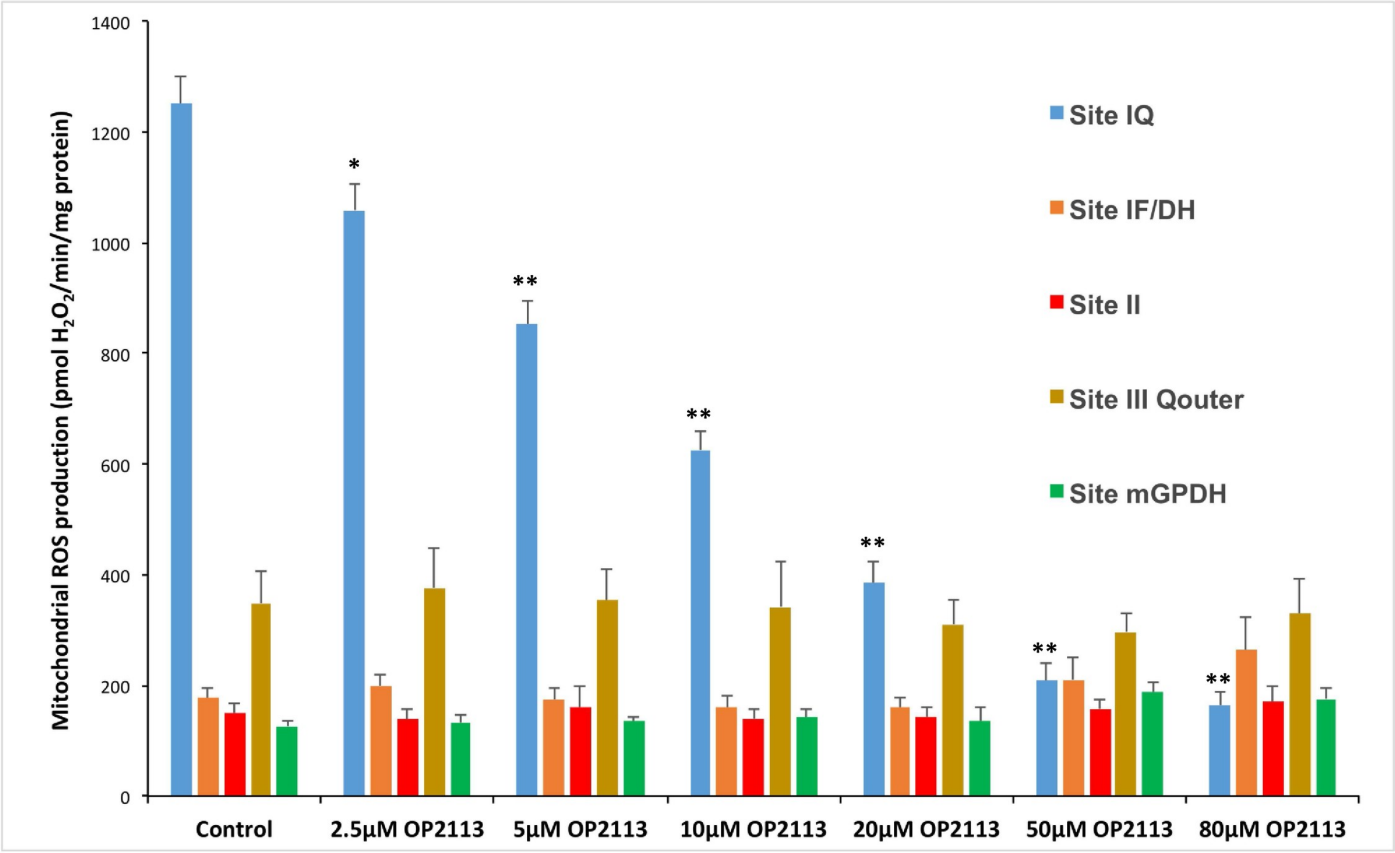
Specific effects of OP2113 on ROS/H_2_O_2_ production at different sites in isolated rat heart mitochondria. The rates of ROS/H_2_O_2_ production were measured under conditions specifically designed by MD brand's group for the identification of the different mitochondrial ROS production sites [21, 22, 24, 38](see Methods section for details). The effects of increasing concentrations of OP2113 (from 2.5 to 80 μM) were tested for each condition of ROS/H_2_O_2_ production. Data are based on 3 to 5 independent experiments, each performed in duplicate or triplicate. *P < 0.01, **P < 0.005 *versus* control group.

Given that OP2113 surprisingly did not inhibit all mitochondrial ROS/H_2_O_2_ production, further investigations were conducted to obtain better insight into the action of the compound on the different mitochondrial sites based on the excellent pioneering work of MD Brand's group [21, 22, 24, 26, 38]. These experiments confirmed that OP2113 affects complex I ROS production, without any measurable effect on the other main sites of ROS/H_2_O_2_ production tested (Fig 4). These results allowed the calculation of a half maximal effective concentration (EC50) of 10.2 ± 0.9 μM for OP2113 on the inhibition of ROS/H_2_O_2_ production by complex I in isolated rat heart mitochondria under our experimental conditions.

The specific effect of OP2113 on a unique site of mitochondrial ROS/H_2_O_2_ production is not only surprising but raises interesting questions about the mechanism of action of OP2113 on mitochondria. These results exclude the hypothesis that the therapeutic effects of OP2113 arise from a mere radical scavenger property as previously reported [28–30]. Indeed, we show in this study that OP2113 does not trap all the ROS produced by mitochondrial respiratory chain, independently of their site of production.

Although the mechanism of action requires further investigations, evidence is presented here that OP2113 directly interferes with mitochondrial complex I ROS production and selectively inhibits superoxide production from the ubiquinone-binding site of complex I (site I_Q_) with no effect on superoxide production from other sites or on oxidative phosphorylation processes. As can be seen in Fig 4, the half maximal effective concentration of OP2113 inhibiting mitochondrial Complex I driven ROS production is equal to 10 μM. This concentration corresponds to 100 nmol /mg protein, an OP2113 quantity that is affecting neither the rotenone-sensitive NADH oxidase activity (see above) nor the oxidative phosphorylation in our experimental conditions (see Fig 1).

Another compound also inhibiting mitochondrial complex I ROS production, N-cyclohexyl-4-(4-nitrophenoxy) benzenesulfonamide, has very recently been described by Brand's group [7] along with other molecules [38, 39]. Similarly with OP2113, these chemicals (S1QELs) do not modify the activity of complex I-driven oxidative phosphorylation [38, 39].

The specificity of OP2113 for mitochondrial ROS production by complex I was further tested *in vitro* on the ROS/H_2_O_2_ production by NAD(P)H oxidase (S2 File). We did not observe any inhibition of resorufin production under these conditions, indicating that OP2113 does not interfere either with the ROS/H_2_O_2_ measurement system (amplex red) or by direct interaction with H_2_O_2_. These results also suggest that OP2113 may not inhibit ROS/H_2_O_2_ production by cytosolic NAD(P)H oxidases, which are likely the major non-mitochondrial ROS/H_2_O_2_ producers in the cells. These results are in striking contrast with previous assertions on the putative effect of OP2113 as a radical scavenger [28–30].

### OP2113 protects heart form ischemia-reperfusion injury (infarct model)

The role of mitochondrial ROS production in ischemia reperfusion injury is now heavily documented and complex I appears to play a central role, during both ischemia and reperfusion [40]. Recent works [13] have for instance demonstrated that the mechanism by which extensive ROS generation occurs at reperfusion involves reverse electron transport at mitochondrial complex I [38] is due to succinate accumulation during ischemia [13, 41], although we did question this mechanism in the context of ischemic preconditioning [42, 43]. It appears also that mitochondrial respiratory chain—and specifically complex I—damages occur during ischemia [40, 44], and that these damages were paralleled by further ROS production and infarct development. Indeed, previous works have shown that a ROS production sensitive to complex I inhibitors occurs during the ischemic phase that may be involved in the mechanisms of heart pharmacological protection [45–48]. Interestingly, ROS production sensitive to complex I inhibitors has been shown to be involved in the damages to complex I occurring during ischemia [44] which increase complex I capacity of ROS production [49] at reperfusion. Reversible ischemia-induced conformational change of complex I to a deactive form has also been shown [50].

Considering the crucial role of ROS from complex I in the complex mechanisms of heart ischemia-reperfusion and the effect of OP2113 on complex I driven ROS production, we appraised that protection of the infarcted heart from ischemia-reperfusion damage may represent a demonstrative experiment to test the effect of OP2113 on mitochondria in living tissues.

**Fig 5:**
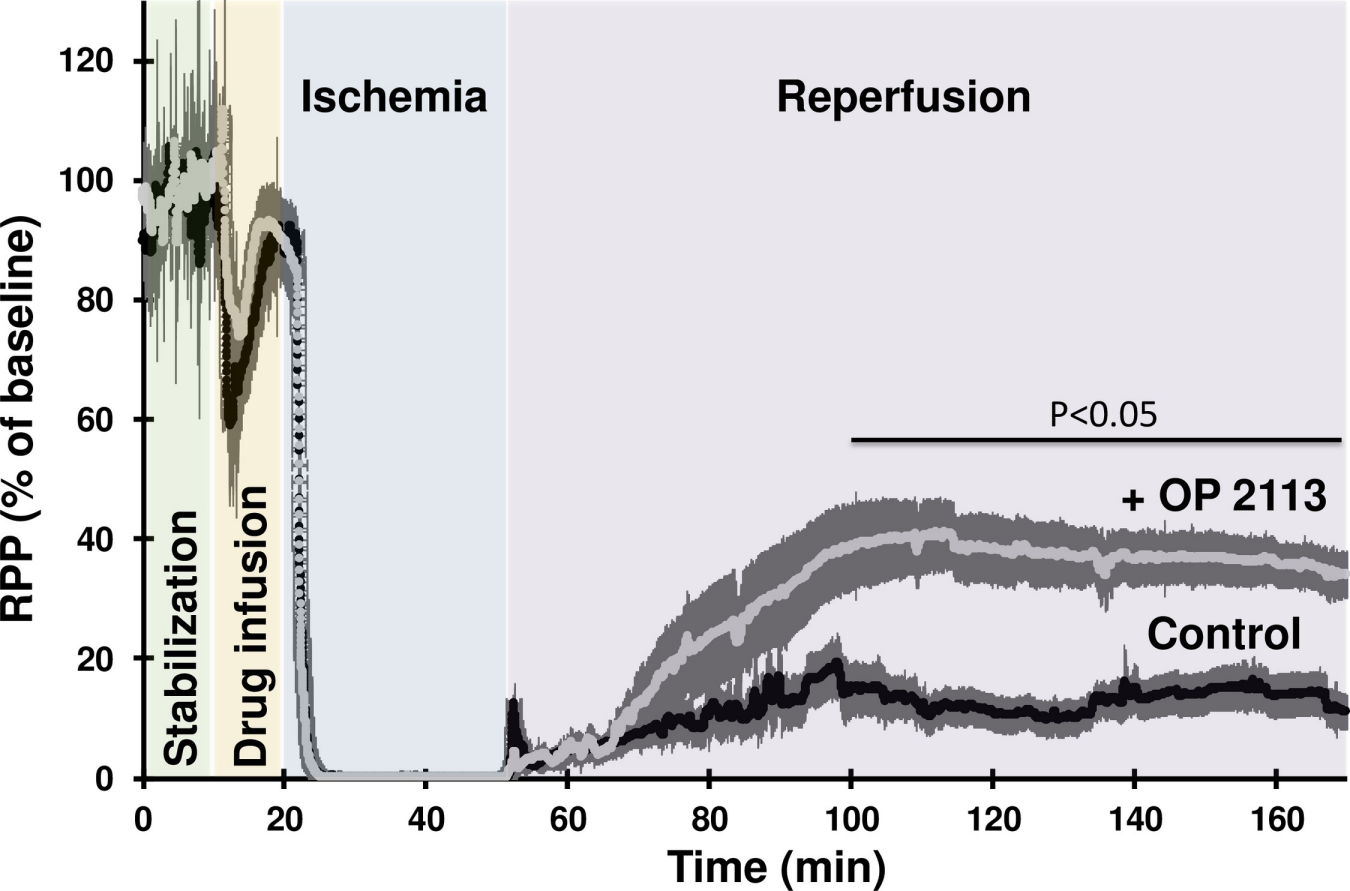
OP2113 improves the recovery of the contractile performance of Langendorff-perfused rat heart during the reperfusion phase following ischemia. This figure shows the time course of the rate-pressure product of two groups of isolated langendorff-perfused rat hearts (n = 6 in each group) during a protocol of ischemia-reperfusion. Rate Pressure Product (RPP), the product of the left ventricular developed pressure (mmHg/beat) by heart rate (beat/min) is used as an index of contractile performance and is expressed as % of baseline value measured at the end of the stabilization period (control 28932±2467 mmHg/min, OP2113 31653±4611 mmHg/min). Each heart was allowed to stabilize during 10 min (green background) before perfusion of vehicle (control group, black trace) or 10 μM OP2113 (+ OP2113 group, grey trace) during 10 more minutes (orange background). Hearts were then submitted to 30 min zero-flow ischemia (blue background) before 120 min of reperfusion in the absence of vehicle or OP2113 (purple background). For more details on the perfusion protocol see also the Methods section and S3 File. Data are expressed as the mean ± SEM for 6 independent experiments. The thickness of the line represents the error bars.

**Fig 6:**
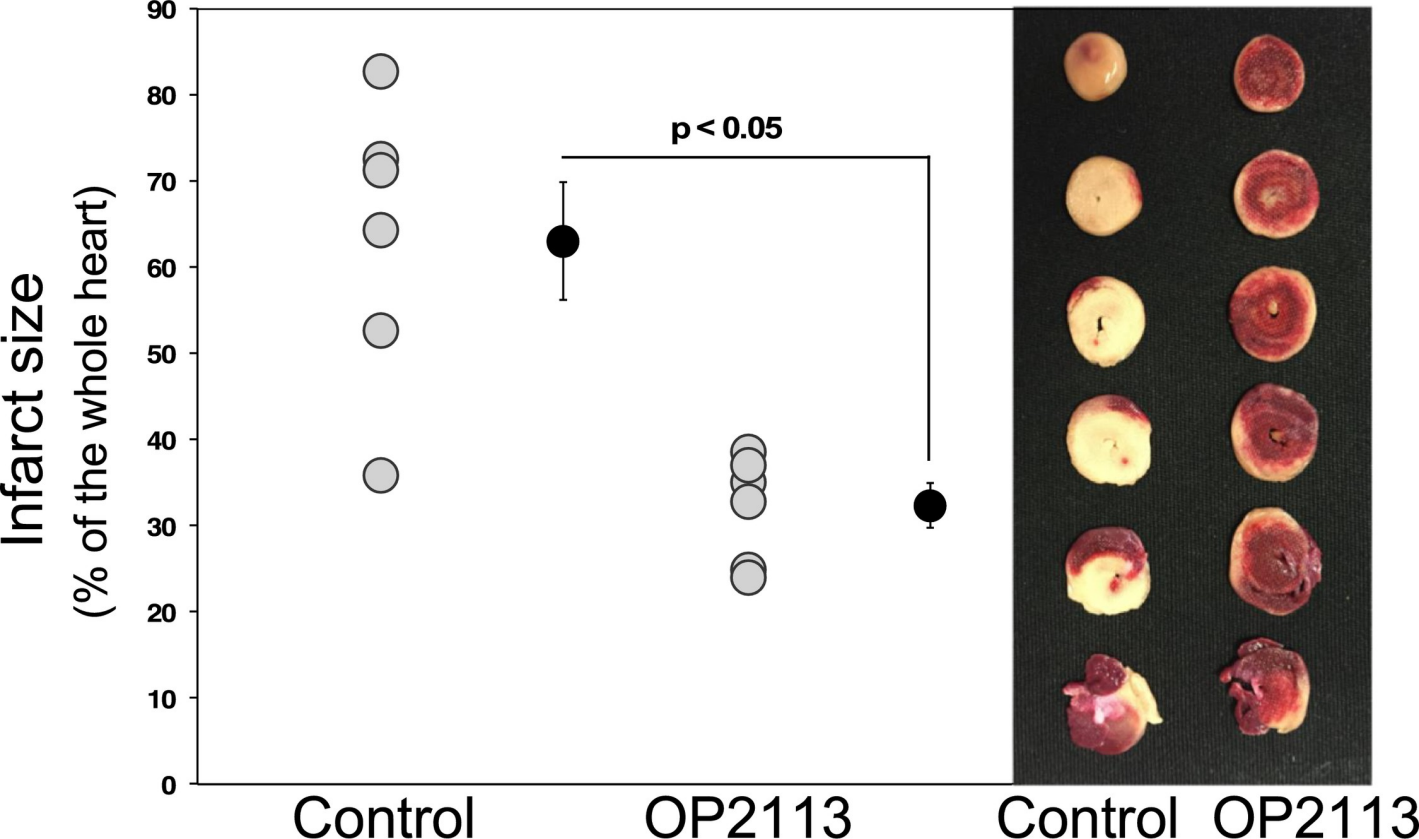
Effect of OP2113 on infarct size. Hearts were pre-treated (OP2113) or not (Control) with OP2113 before 30 min of ischemia followed by 120 min of reperfusion (see Fig 5). Left panel, grey dots represent individual experiments and black dots mean ± SEM (see Methods, n = 6 independent experiments for each condition). Right panel, typical pictures obtained in the control and OP2113 groups following TTC staining. The tissues appearing in red are living tissues stained with TTC, whereas damaged tissue appears white.

This hypothesis was assessed by investigating the cardioprotective effects of OP2113 pre-treatment of Langendorff-perfused rat hearts submitted to an ischemia-reperfusion protocol (see S3 File). Cardioprotection was assessed from the recovery of contractile performance during reperfusion (Fig 5) and quantification of infarct size by triphenyltetrazolium chloride (TTC) staining at the end of the reperfusion period (Fig 6). Fig 5 shows the time course of the rate-pressure product (RPP calculated as the product of left ventricular developed pressure by heart rate). After the stabilization period, the presence of ethanol in the vehicle during the infusion period caused a similar transitory decrease of the RPP in the two groups studied (Fig 5, orange background). Contraction was stopped during the 30 min ischemia and restart at reperfusion. Fig 5 shows that pre-treatment with OP2113 significantly improves the recovery of RPP at the end of the reperfusion period (34% *vs* 11% of baseline value in OP2113 and control conditions, respectively, p°<°0.05).

The infarct size was analyzed by TTC staining and quantified at the end of the reperfusion period for a series of 6 experiments per condition (see typical photographs of heart slices in Fig 6 right panel). Results clearly demonstrate that OP2113 significantly decreased infarct size at the end of reperfusion by approximately 50% (Fig 6, left panel). This observation fits well with the better recovery of heart contractile performance of hearts treated with OP2113 as compared to controls (see Fig 5).

## Discussion

When tested on isolated mitochondria from rat heart, OP2113 effectively decreases mitochondrial ROS/H_2_O_2_ production (in isolated mitochondria, H_2_O_2_ is produced from the reduction of superoxide anion by mitochondrial superoxide dismutase). Moreover, the results presented here clearly demonstrate that OP2113 presents a very strong selectivity towards the formation of ROS by site I_Q_ in complex I (Fig 1), which demonstrates that OP2113 does not simply interact with superoxide radicals but specifically prevents their formation by complex I. In that respect, OP2113 therefore appears as a member of the brand new class of oxidative stress protectants. Whereas antioxidants generally do not interfere directly with electron transport and scavenge ROS and/or H_2_O_2_ downstream from production and therefore can never fully suppress the effect of ROS [7], OP2113 may act differently by preventing ROS formation and thus being more active to protect mitochondria from their own ROS. Data presented here further demonstrate that OP2113 is a specific inhibitor of ROS formation at site I_Q_ of complex I of the mitochondrial respiratory chain. Further experiments are however required to ascertain whether OP2113 has no effect on other mitochondrial ROS production sites, but this does not preclude the above conclusions. Incidentally, the observed inhibition of the rotenone-sensitive NADH oxidase activity in broken mitochondria may reflect a different process, since it occurs at higher concentrations than ROS inhibition and without any effect on oxidative phosphorylation. Nonetheless, at this stage we cannot rule out that in our *ex vivo* experiments the cardioprotective effect brought by OP2113 could be secondary to complex I inhibition during the ischemic phase, a cardioprotective strategy as shown by Lesnefsky and collaborators [44, 45, 51].

We also present evidence that OP2113 may interact only with mitochondria without affecting ROS formation in the cytosol and therefore would not affect intracellular signaling. Selective modulators of superoxide production from site I_Q_ would offer unique opportunities to probe the putative role of mitochondrial ROS production in normal and pathological processes [7] and during a life span [2]. OP2113’s specificity towards mitochondrial ROS production ("bad" ROS) would preserve cytosolic ROS signaling ("good" ROS) and therefore appears as a very promising property that may circumvent the bias of the use of non-specific antioxidants in clinical trials.

Since OP2113 does not present a permanent positive charge, it may not accumulate in mitochondria due to the inner mitochondrial membrane potential difference. However, to our knowledge, all previous chemical and pharmacological data [31] confirm the high lipophilicity of the molecule and its large distribution in tissues. The observed cardioprotective effect of OP2113 against damages induced by ischemia-reperfusion strongly suggest that, at least when added before reperfusion, the drug reaches mitochondrial membranes.

As discussed previously, the mechanisms leading to ischemia-reperfusion injury are highly complex and ROS, especially from complex I, are involved during both ischemia and reperfusion [40]. At this stage, we cannot effectively conclude if OP2113 acts by protecting complex I from his own ROS during the ischemic period [44–49] or by inhibiting reverse electron transfer and consequent ROS production at the onset of reperfusion [13, 38, 41]. Furthermore, as underlined in the introduction, besides these new specific properties, we must also consider the role of OP2113 as a potential H_2_S donor [27, 32–34] in the isolated heart ischemia-reperfusion experiments. Indeed H_2_S is now considered as a signaling molecule with potential therapeutic applications [52–54], including ischemia-reperfusion and cardiac pathologies [55]. H_2_S at low concentration may be oxidized by mammalian mitochondria [56] and protect mitochondria during the ischemic phase [40] and reperfusion [54, 57]. These mechanisms may also be involved in heart protection by OP2113 during ischemia-reperfusion (Fig 6).

**Fig 7:**
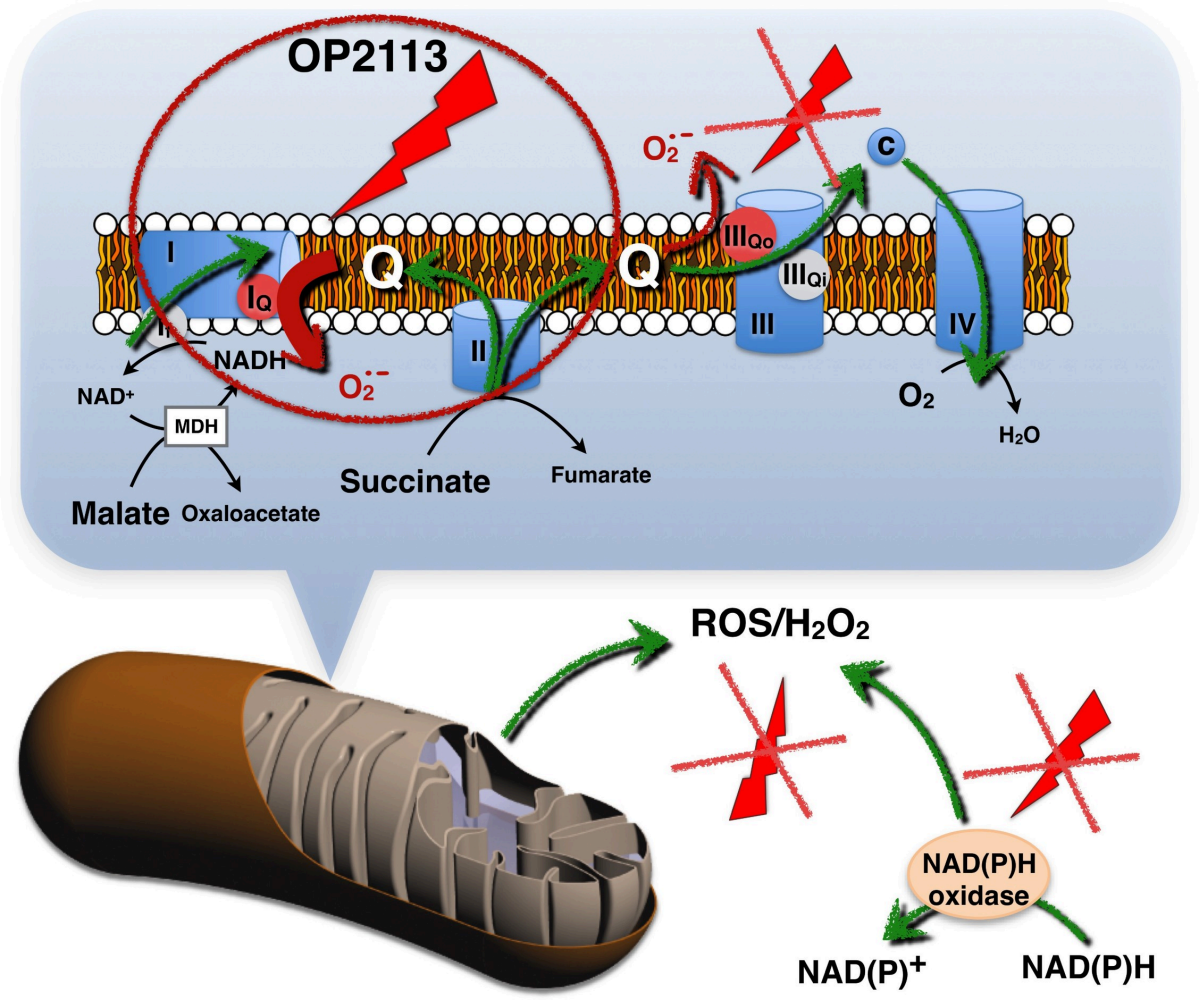
Scheme presenting the specific effects of OP2113 on ROS production by complex I.

In summary, OP2113 acts upstream from ROS production, therefore insuring increased protection compared with standard antioxidants. OP2113 acts specifically on mitochondrial ROS production to ensure mitochondrial protection, and this action is crucial for numerous pathologies, especially cardiac diseases. OP2113 does not seem to interfere with cell signaling ("good" ROS) (Fig 7). OP2113 acts specifically on site I_Q_ in complex I, which is the main mitochondrial site and may be implicated in important diseases, including Parkinson's and cardiac arrhythmias.

To conclude, OP2113’s properties may represent a break-through in the search for specific modulators of ROS/H_2_O_2_ production in cells. This is a timely and important discovery given that OP2113 has a great advantage over newly discovered molecules as it is currently authorized for use in humans [31] and may therefore be rapidly included in clinical trials [58–60]. OP2113 may represent the first medicinal member of a new class of "protectants" that specifically prevent ROS production inside mitochondria and may therefore be used for mitochondrial protection during various oxidative stresses, therefore preventing diseases with minimal effects on crucial cellular ROS signaling.

## Methods

### Animal procedures and ethics statement

All experiments described adhered to the guidelines in the National and European Research Council Guide for the care and use of laboratory animals. P. Diolez has a valid license to conduct experiments on animals from the Service Vétérinaire de la Santé et de la Protection Animale of the Ministère de l’agriculture et de la Forêt, France (03/17/1999, license number 3308010). All procedures conformed to the UK Animals (Scientific Procedures) Act 1986 and the Guide for the Care and Use of Laboratory Animals published by the National Institutes of Health (NIH Publication No. 85–23. revised 1996).

### Materials

All the chemicals were reagent grade and purchased from Sigma Chemical (St. Louis, MO), except for sucrose and NADH oxidase (Merck, Darmstadt, Germany). OP2113 [anetholtrithion, 5-(4-Methoxyphenyl)-3H-1,2-dithiole-3-thione, CAS 532-11-6] was a gift from the private company OP2 drugs (Pessac, France). A 15 mM stock solution of OP2113 was prepared in DMSO and stored away from light at 0°C for only few days. For heart perfusion a daily prepared solution was used.

### Isolation of mitochondria

Male Wistar rats (250–325 g; obtained from Janvier Labs, Le Genest-Saint-Isle, France) were anesthetized using 3% isoflurane, heparinized and euthanized by a lethal intra-peritoneal injection of pentobarbital (130 mg/kg), and the heart was quickly removed and washed in cold isolation medium containing 100 mM sucrose, 180 mM KCl, 50 mM Tris, 5 mM MgCl2, 10 mM EDTA, and 0.1% (w/v) defatted BSA (pH 7.2).

Isolation of heart mitochondria was performed in a cold chamber. Before homogenization, hearts (approximately 1.5 g) were minced with scissors and treated for 5 min in 5 ml of the same medium supplemented with protease (2 mg of bacterial proteinase type XXIV per ml of isolation buffer) with stirring. The tissue suspension was poured into a 50-ml glass Potter homogenizer, diluted with 20 ml of isolation buffer, and then homogenized for 3 min using a motorized Teflon pestle. The homogenate was filtered through bolting cloth (Sefar Nitex) to remove debris and centrifuged at 8,000 g for 10 min. The resulting pellet was rinsed with 5 ml of isolation buffer, resuspended in 25 ml of the same buffer, and then subjected to low speed centrifugation (400 g) for 8 min. The resulting supernatant was centrifuged twice at 7,000 g for 15 min to yield a washed mitochondrial pellet that was gently resuspended in 150 μl of isolation buffer. Protein concentration was determined by the Bradford method (Sigma, kit # B6916) using BSA as a standard. Mitochondria were kept on ice at a final concentration of 40–50 mg/ml for less than 5 hours.

### Mitochondrial respiration

Oxygen consumption rates of heart mitochondria (0.1 mg/ml) incubated in the absence or presence of OP2113 at increasing doses (from 0 to 80 μM final concentration) were recorded polarographically under constant stirring at 25°C using a high resolution oximeter (Oxygraph-2K, Oroboros Instruments, Austria). The respiration medium consisted of 140 mM sucrose, 100 mM KCl, 1 mM EGTA, 20 mM MgCl_2_, 10 mM KH_2_PO_4_, and 1 g/L (w/v) BSA essentially fatty acid free (pH 7.2). Oxidative phosphorylation has been carried out using various substrate combinations: Glutamate (5 mM)/malate (2.5 mM) as complex I substrates, succinate (5 mM in the presence of 1.5 μM rotenone) as complex II substrates, and the combination Glutamate + Malate + Succinate.

### Mitochondrial ROS/H_2_O_2_ production

Rates of ROS/H_2_O_2_ production from heart mitochondria were assessed through the oxidation of the colorless, non-fluorescent indicator Amplex Red in the presence of exogenous horseradish peroxidase (HRP, EC 1.11.1.7, Sigma). H_2_O_2_ reacts with Amplex Red in a 1:1 stoichiometry, yielding the fluorescent compound resorufin (excitation: 560 nm; emission: 585 nm), which is stable once formed. Fluorescence was measured continuously with a spectrofluorometer equipped with temperature control and stirring (SAFAS Xenius, Monaco).

Isolated mitochondria (0.1 mg/ml) were incubated in the same experimental buffer as previously described supplemented with 15 μM Amplex Red and 10 μg/ml HRP. Glutamate (5 mM)/malate (2.5 mM) together with succinate (5 mM) were used as complex I and complex II substrates, respectively. Experiments were conducted under non-phosphorylating conditions in the presence of 15 μM atractyloside (inhibitor of adenine nucleotide translocator), *i*.*e*., under conditions where the mitochondrial membrane potential is maximal. Afterwards, rotenone (1.5 μM), antimycin A (2 μM), and myxothiazol (0.2 μM) were sequentially added to inhibit the redox centers within the electron transfer chain (see Fig 2), namely sites I_Q_, I_F_ (with rotenone), III_Qi_ (with antimycin A) and III_QO_ (with myxothiazol). The assay was finally calibrated with known amounts of H_2_O_2_ (steps of 300 nM) in the presence of all relevant compounds, including OP2113. The control test of the absence of effect of OP2113 on the amplex red assay itself and NAD(P)H oxidase ROS/H_2_O_2_ production was performed in the absence of cardiac mitochondria and the presence of NAD(P)H oxidase (EC 1.6.3.3, 1 mU/ml) and NADH (150 μM) solutions.

The measurement of the rates of ROS/H_2_O_2_ production from major separate mitochondrial sites was performed as described by MD Brand's group [21, 24]. Sites of mitochondrial superoxide/H_2_O_2_ production were targeted individually using distinct combinations of mitochondrial substrates and inhibitors [21, 24] designed to generate maximal rates of ROS/H_2_O_2_ production predominantly from a single site within the respiratory chain. The sites of production targeted and the solutions used to drive H_2_O_2_ production were as follows: site I_Q_ with 5 mM succinate; site I_F_/DH with 5 mM glutamate + 2.5 mM malate and 4 μM rotenone; site III_Qo_ with 5 mM succinate, 4 μM rotenone and 2.5 μM antimycin A; site II_F_ with 15 μM palmitoylcarnitine, 2.5 μM antimycin A and 2 μM myxothiazol; and mitochondrial glycerol-3-phosphate dehydrogenase (mGPDH) with 25 mM glycerolphosphate, 4 μM rotenone, 2.5 μM antimycin A, 1 mM malonate and 2 μM myxothiazol.

The final effect of the OP2113 compound was scaled to positive controls included in each assay. In addition, 4 μM CCCP, 20 mM aspartate, 2 μM myxothiazol, and 10 mM malonate were used as positive controls for site I_Q_, I_F_, III_Qo_ and II_F,_ respectively [21, 24]. No positive control is available in the case of mGPDH.

Results were analyzed using one-way analysis of variance, followed by Bonferroni’s test to check for significant differences, using Statview Software. Significance was accepted at P < 0.05.

### Heart perfusion

Male Wistar rats (250–300 g) were anesthetized by 3% isoflurane, heparinized and euthanized by a lethal IP injection of pentobarbital (130 mg/kg). Hearts (~0.95 g fresh weight) were rapidly harvested and placed into ice-cold Krebs-Henseleit buffer containing (in mmol/L): NaCl 118, NaHCO_3_ 25, KCl 4.8, KH_2_PO_4_ 1.2, MgSO_4_ 1.2, glucose 11 and CaCl_2_ 1.8. The solution was gassed with 95% O_2_/5% CO_2_ at 37°C (pH 7.4). Langendorff heart perfusions were performed as described previously [61], and isometric contractile performance (rate-pressure product (RPP)) was assessed from continuous monitoring of the left ventricular developed pressure (LVDP) via a balloon placed in the left ventricle and connected to a pressure transducer (RPP (mmHg/min) = LVDP (mmHg/beat) x heart rate (beat/min)) [61]. Hearts were perfused in a constant flow mode (12 ml/min) during 10 min for stabilization followed by 10 min treatment with the vehicle (Control; final concentrations: 0.83% ethanol + 0.07% Dimethyl-Sulfoxide) or 10 μM OP2113 solution in the same vehicle. Global normothermic ischemia was induced by halting perfusion flow for 30 min while immersing the heart in perfusion buffer thermostabilized at 37°C. Then, hearts were reperfused for 2 hours.

### Assessment of infarct size

At the end of the 2-hour reperfusion period, hearts were stained with triphenyltetrazolium chloride (TTC). Hearts were stained by perfusion for 7 min at 12 ml/min with a 1% (w / v) TTC solution. Hearts were then detached from the cannula and incubated for 4 min at 37°C before being sliced perpendicularly to the longitudinal axis into 6 slices. The slices were then treated in 4% (w/v) formalin solution overnight at 4°C and weighed before both sides of each slice were photographed. The surface of the necrotic and at risk areas of each side were determined for each photograph by planimetry (AlphaEase v5.5). Infarct size was expressed as the percentage of the total cross-sectional area of the heart given that the total heart was subjected to ischemia under our conditions. Data in supporting information S3 File.

### Statistical analysis

Data from 6 independent heart perfusions are expressed as the means ± SEM. As the number in each group was less than 20, the distribution was considered non-normal. Consequently, a non-parametric Mann-Whitney test (SPSS statistics 17.0) was performed for comparisons between the control and OP2113 groups. The results were considered statistically significant if the *p*-value was less than 0.05.

## Supporting information

S1 File Effect of OP2113 on mitochondrial NADH oxidase activity. After freeze-thaw treatment, rat heart mitochondria were used to assess mitochondrial rotenone-sensitive NADH oxidase activity by polarography as described in the supplementary Materials and Methods. Panel A: Typical polarographic trace showing the rotenone-sensitive NADH oxidase activity and the effect of the addition of increasing quantity of OP2113 from 50 to 800 nmol / mg mitochondrial protein on oxygen consumption. Panel B: Bar graph representing the mean oxygen consumption expressed in nmol O_2_ / min / mg mitochondrial protein. Rotenone addition completely stop oxygen consumption suggesting that the activity is mainly supported by the mitochondrial complex I. Data are presented as means ± SD. 4 independent mitochondrial preparation were used for the assay and for each mitochondrial batch the assay was realized in quadruplicate. High quantity of OP2113 inhibit partly the mitochondrial rotenone-sensitive NADH oxidase activity.
(ZIP)

S2 File Effect of OP2113 on the artificial H_2_O_2_-producing NAD(P)H oxidase system. The rates of H_2_O_2_ production were measured in the presence of NAD(P)H oxidase (1 mU/ml) and NADH (150 μM), and in the absence of heart mitochondria. Data are based on 3 independent experiments, each performed in duplicate. No significant effect of OP2113 on this experimental H_2_O_2_ production was noted.
(ZIP)

S3 File Detailed information and pictures about ischemia/reperfusion in rat heart. Supporting data contain supplementary informations concerning the experiments on isolated rat heart ischemia and reperfusion. Raw data presents contractile activity (RPP), whole heart oxygen consumption (MVO_**2**_) during the pre-schemic and post-ischemic (reperfusion) phases for all the experiments, as well as all data used for the determination of infarct size. Separate files describe the results of all the statistical analyses presented in Figs 5 and 6. Finally, supplementary figures present pre-ischemic RPP and MVO_2_ and reperfusion phases (MVO_2_ and RPP to MVO_2_ ratio), as well as a graphic description of the protocols used in the study.
(ZIP)

## Abbreviations

ATR: atractyloside
BSA: bovine serum albumin
CCCP: Carbonylcyanure m-chlorophénylhydrazone
DMSO: dimethyl sulfoxide
HRP: Horseradish peroxidase
LVDP: Left ventricular developed pressure
mGPDH: mitochondrial glycerol-3-P dehydrogenase
RET: reverse electron transfer
ROS: reactive oxygen species
RPP: rate-pressure product
S1QELs: Suppressors of complex 1 site Q electron leak
TTC: Triphenyl tetrazolium chloride
