# Supplementary material for: An old medicine as a new drug to prevent mitochondrial complex I from producing oxygen radicals
Source: PLoS One. 2019 May 2;14(5):e0216385. doi: 10.1371/journal.pone.0216385 (PMC6497312; doi:10.1371/journal.pone.0216385)
Supplement: S1 File — After freeze-thaw treatment, rat heart mitochondria were used to assess mitochondrial rotenone-sensitive NADH oxidase activity by polarography as described in the supplementary Materials and Methods. Panel A: Typical polarographic trace showing the rotenone-sensitive NADH oxidase activity and the effect of the addition of increasing quantity of OP2113 from 50 to 800 nmol / mg mitochondrial protein on oxygen consumption. Panel B: Bar graph representing the mean oxygen consumption expressed in nmol O2 / min / mg mitochondrial protein. Rotenone addition completely stop oxygen consumption suggesting that the activity is mainly supported by the mitochondrial complex I. Data are presented as means ± SD. 4 independent mitochondrial preparation were used for the assay and for each mitochondrial batch the assay was realized in quadruplicate. High quantity of OP2113 inhibit partly the mitochondrial rotenone-sensitive NADH oxidase activity. (ZIP) [file pone.0216385.s001.zip › NADH oxidase (S1)/Supplemental Legend to S1 Fig.docx]

***Supplementary Figure 1. Mitochondrial NADH oxidase activity.***

Broken rat heart mitochondria were used to assess mitochondrial NADH oxidase activity by polarography as described in the supplementary Materials and Methods. **Panel A:** Typical polarographic trace showing the NADH oxidase activity (rotenone sensitive) and the effect of the addition of increasing quantity of OP2113 from 50 to 800 nmol / mg mitochondrial protein on oxygen consumption. **Panel B:** Bar graph representing the mean oxygen consumption expressed in nmol O_2_ / min / mg mitochondrial protein obtained in the different experimental conditions. Rotenone addition completely stop oxygen consumption suggesting that the activity is mainly supported by the mitochondrial complex I. Dare are presented as means ± SD. 4 independent mitochondrial preparation were used for the assay and for each preparation the assay was realized in quadruplicate.
