# Supplementary material for: An old medicine as a new drug to prevent mitochondrial complex I from producing oxygen radicals
Source: PLoS One. 2019 May 2;14(5):e0216385. doi: 10.1371/journal.pone.0216385 (PMC6497312; doi:10.1371/journal.pone.0216385)
Supplement: S1 File — After freeze-thaw treatment, rat heart mitochondria were used to assess mitochondrial rotenone-sensitive NADH oxidase activity by polarography as described in the supplementary Materials and Methods. Panel A: Typical polarographic trace showing the rotenone-sensitive NADH oxidase activity and the effect of the addition of increasing quantity of OP2113 from 50 to 800 nmol / mg mitochondrial protein on oxygen consumption. Panel B: Bar graph representing the mean oxygen consumption expressed in nmol O2 / min / mg mitochondrial protein. Rotenone addition completely stop oxygen consumption suggesting that the activity is mainly supported by the mitochondrial complex I. Data are presented as means ± SD. 4 independent mitochondrial preparation were used for the assay and for each mitochondrial batch the assay was realized in quadruplicate. High quantity of OP2113 inhibit partly the mitochondrial rotenone-sensitive NADH oxidase activity. (ZIP) [file pone.0216385.s001.zip › NADH oxidase (S1)/Supplemental Methods S1 Fig.docx]

**Measurement of mitochondrial NADH oxidase activity by polarography**

The NADH oxidase assay was adapted from Hoppel et al. [[1](#_ENREF_1)]. The assay was designed to assess the effect of 50, 200 and 800 nmol of OP2113 per mg of mitochondrial protein on rotenone sensitive NADH oxidase activity. These concentrations correspond to the concentrations of 5, 20 and 80 µM used for the assays of the effects of OP2113 on oxidative phosphorylation (Fig 1 of the main manuscript).

Frozen-thawed heart mitochondria were solubilized at a final concentration of 10 mg / ml in a buffer containing, 0.1 mol / L of KH_2_PO_4_, 1% (W / V) deoxycholate, pH 7.2 with KOH at room temperature. Broken mitochondria (50 µg) were used to assess the rotenone-sensitive NADH oxidation by polarography (Oxygraph-2K, Oroboros Instruments, Austria) in an assay buffer containing 20 mmol / L of KH_2_PO_4_, 0.1 mmol / L EDTA, 0.1 % (W / V) of BSA essentially fatty acid free, pH 7.4 with KOH at room temperature. The assay was performed as follow: addition of 2 ml of the assay buffer, supplementation with 32 µmol / L of Cytochrome c, addition of mitochondria (50 µg final). After closing the oxygraph chamber, the reaction was started by the addition of 2.8 mmol / L of NADH as shown in the panel A of the supplemental figure 3 (S3 Fig). Then, the different concentrations of OP2113 were added before the addition of rotenone (5 µmol / L final) at the end of the assay. Rotenone stopped nearly 100% of the oxygen consumption, demonstrating that on isolated rat heart mitochondria, NADH oxidase activity is mainly supported by complex I.

1. Hoppel CL, Kerr DS, Dahms B, Roessmann U. Deficiency of the reduced nicotinamide adenine dinucleotide dehydrogenase component of complex I of mitochondrial electron transport. Fatal infantile lactic acidosis and hypermetabolism with skeletal-cardiac myopathy and encephalopathy. J Clin Invest. 1987 Jul;80(1):71-7. PubMed PMID: 3110216. Pubmed Central PMCID: PMC442203.
