# Supplementary figures and images for: An old medicine as a new drug to prevent mitochondrial complex I from producing oxygen radicals
Source: PLoS One. 2019 May 2;14(5):e0216385. doi: 10.1371/journal.pone.0216385 (PMC6497312; doi:10.1371/journal.pone.0216385)

## Slide 1
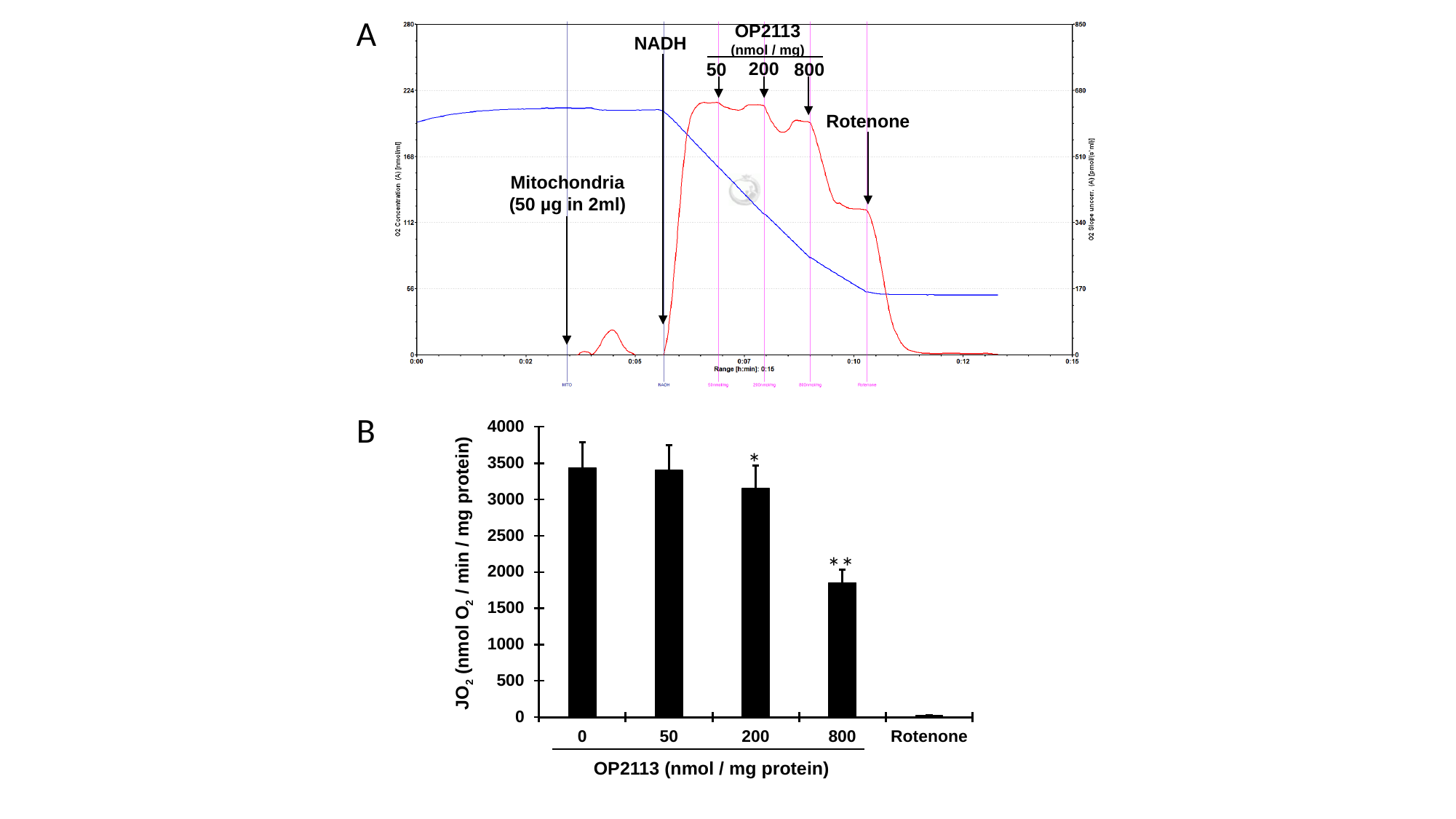

A
OP2113
(nmol / mg)
NADH
200
800
50
Rotenone
Mitochondria
(50 µg in 2ml)
B
*
**
JO2 (nmol O2 / min / mg protein)
OP2113 (nmol / mg protein)

Supplement: S1 File — After freeze-thaw treatment, rat heart mitochondria were used to assess mitochondrial rotenone-sensitive NADH oxidase activity by polarography as described in the supplementary Materials and Methods. Panel A: Typical polarographic trace showing the rotenone-sensitive NADH oxidase activity and the effect of the addition of increasing quantity of OP2113 from 50 to 800 nmol / mg mitochondrial protein on oxygen consumption. Panel B: Bar graph representing the mean oxygen consumption expressed in nmol O2 / min / mg mitochondrial protein. Rotenone addition completely stop oxygen consumption suggesting that the activity is mainly supported by the mitochondrial complex I. Data are presented as means ± SD. 4 independent mitochondrial preparation were used for the assay and for each mitochondrial batch the assay was realized in quadruplicate. High quantity of OP2113 inhibit partly the mitochondrial rotenone-sensitive NADH oxidase activity. (ZIP) [file pone.0216385.s001.zip › NADH oxidase (S1)/S1 Fig.pptx]

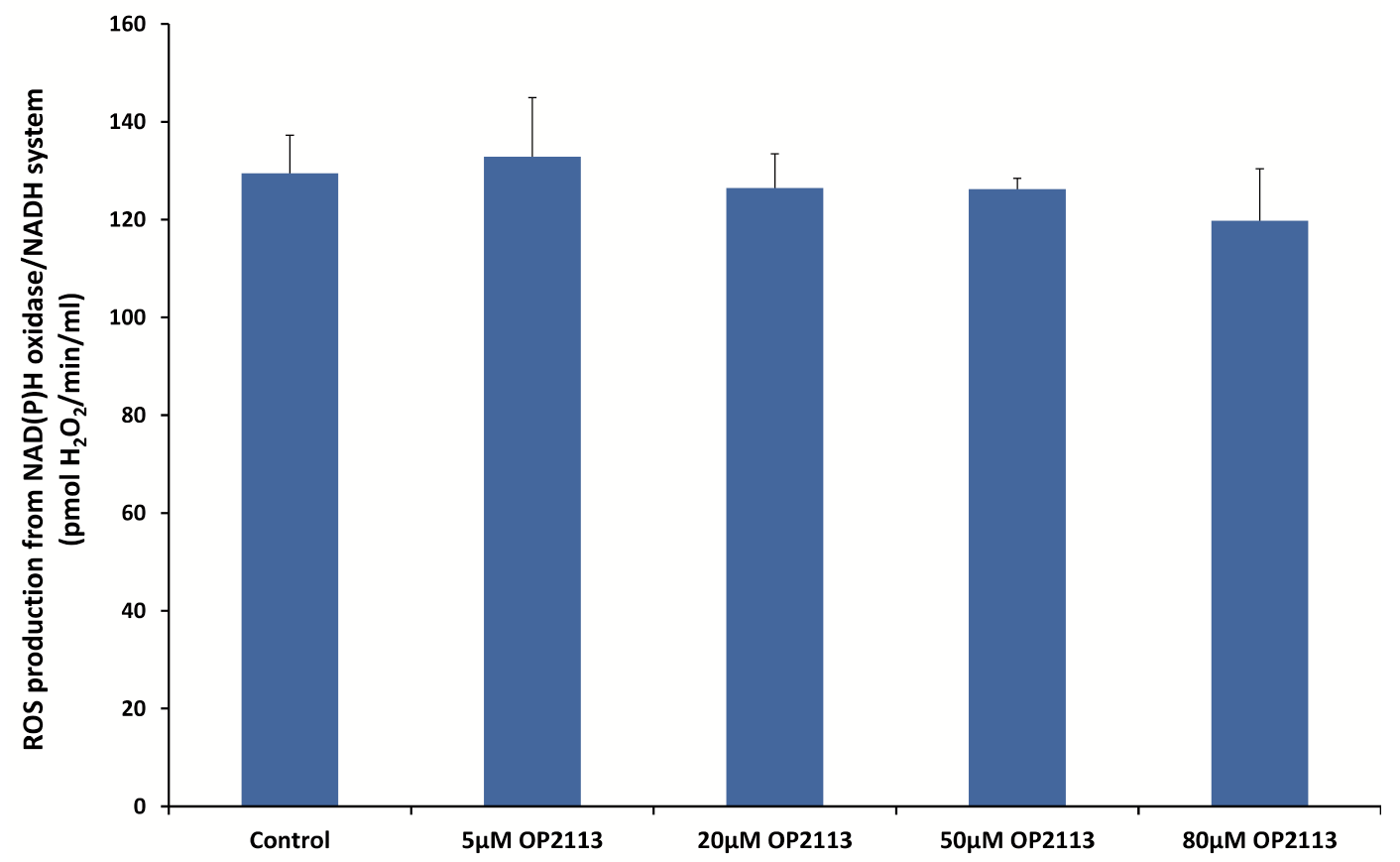

Supplement: S2 File — The rates of H2O2 production were measured in the presence of NAD(P)H oxidase (1 mU/ml) and NADH (150 μM), and in the absence of heart mitochondria. Data are based on 3 independent experiments, each performed in duplicate. No significant effect of OP2113 on this experimental H2O2 production was noted. (ZIP) [file pone.0216385.s002.zip › NAD(P)H oxidase (S2)/S2_Fig.png]
