# Supplementary material for: An old medicine as a new drug to prevent mitochondrial complex I from producing oxygen radicals
Source: PLoS One. 2019 May 2;14(5):e0216385. doi: 10.1371/journal.pone.0216385 (PMC6497312; doi:10.1371/journal.pone.0216385)
Supplement: S2 File — The rates of H2O2 production were measured in the presence of NAD(P)H oxidase (1 mU/ml) and NADH (150 μM), and in the absence of heart mitochondria. Data are based on 3 independent experiments, each performed in duplicate. No significant effect of OP2113 on this experimental H2O2 production was noted. (ZIP) [file pone.0216385.s002.zip › NAD(P)H oxidase (S2)/Supplemental Legend to S2_Fig.docx]

***Supplementary Figure 2. Effect of OP2113 on ROS/H_2_O_2_ production from the NADH-NAD(P)H oxidase system.***

The bar graph shows that increasing concentration of OP2113 up to 80 µM has no effect on H_2_O_2_ production from the NADH-NAD(P)H oxidase system. H_2_O_2_ was detected by the system Amplex Red + peroxidase as described in the corresponding supplemental Materials and Methods. Data are presented as means ± SEM, n=3 independent experiments.
