# Supplementary material for: An old medicine as a new drug to prevent mitochondrial complex I from producing oxygen radicals
Source: PLoS One. 2019 May 2;14(5):e0216385. doi: 10.1371/journal.pone.0216385 (PMC6497312; doi:10.1371/journal.pone.0216385)
Supplement: S2 File — The rates of H2O2 production were measured in the presence of NAD(P)H oxidase (1 mU/ml) and NADH (150 μM), and in the absence of heart mitochondria. Data are based on 3 independent experiments, each performed in duplicate. No significant effect of OP2113 on this experimental H2O2 production was noted. (ZIP) [file pone.0216385.s002.zip › NAD(P)H oxidase (S2)/Supplemental_Material_S2_Fig.docx]

***Supplemental Materials and Methods***

***Measurement of ROS/H_2_O_2_ production from the NADH-NAD(P)H oxidase system***

A model reaction system using NADH and NAD(P)H oxidase was used for generating non-cellular free radicals. As previously described for mitochondrial ROS/H_2_O_2_ assay, the Amplex Red method was applied but the working buffer was deprived of cardiac mitochondria and only contained NADH plus NAD(P)H oxidase (EC 1.6.3.3) at final respective concentrations of 150 µM and 1 mU/ml.
