# Supplementary material for: An old medicine as a new drug to prevent mitochondrial complex I from producing oxygen radicals
Source: PLoS One. 2019 May 2;14(5):e0216385. doi: 10.1371/journal.pone.0216385 (PMC6497312; doi:10.1371/journal.pone.0216385)
Supplement: S3 File — Supporting data contain supplementary informations concerning the experiments on isolated rat heart ischemia and reperfusion. Raw data presents contractile activity (RPP), whole heart oxygen consumption (MVO2) during the pre-schemic and post-ischemic (reperfusion) phases for all the experiments, as well as all data used for the determination of infarct size. Separate files describe the results of all the statistical analyses presented in Figs 5 and 6. Finally, supplementary figures present pre-ischemic RPP and MVO2 and reperfusion phases (MVO2 and RPP to MVO2 ratio), as well as a graphic description of the protocols used in the study. (ZIP) [file pone.0216385.s003.zip › Heart perfusion (S3)/Heart perfusion supplemental figures.pptx]

## Slide 1
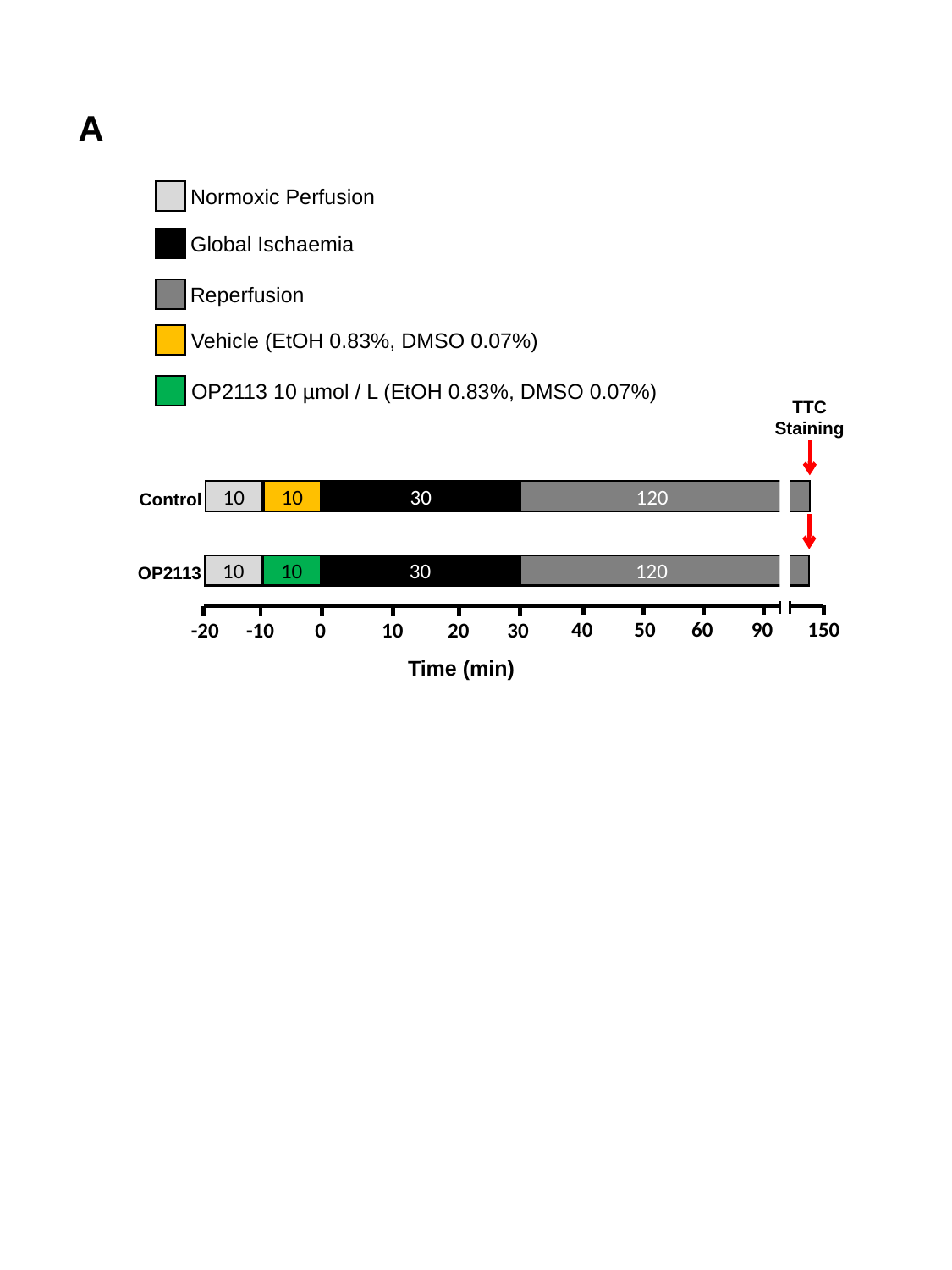

A
Normoxic Perfusion
Global Ischaemia
Reperfusion
Vehicle (EtOH 0.83%, DMSO 0.07%)
OP2113 10 µmol / L (EtOH 0.83%, DMSO 0.07%)
TTC
Staining
10
10
30
120
Control
10
10
30
120
OP2113
40
50
60
90
150
-20
-10
0
10
20
30
Time (min)

## Slide 2
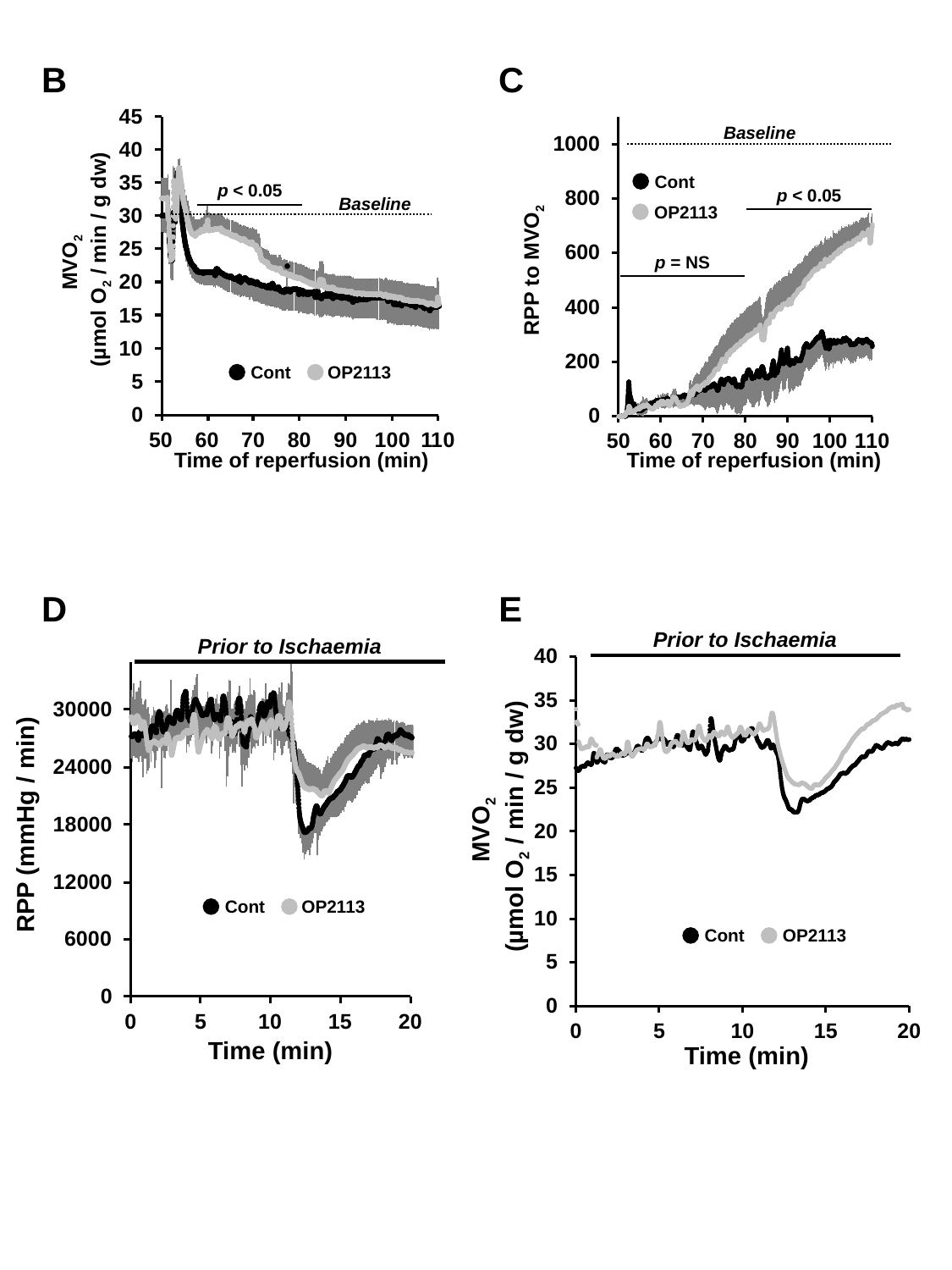

B
C
Baseline
Cont
p < 0.05
p < 0.05
Baseline
OP2113
MVO2
(µmol O2 / min / g dw)
p = NS
RPP to MVO2
Cont
OP2113
Time of reperfusion (min)
Time of reperfusion (min)
D
E
Prior to Ischaemia
Prior to Ischaemia
MVO2
(µmol O2 / min / g dw)
RPP (mmHg / min)
Cont
OP2113
Cont
OP2113
Time (min)
Time (min)
