# Supplementary material for: An old medicine as a new drug to prevent mitochondrial complex I from producing oxygen radicals
Source: PLoS One. 2019 May 2;14(5):e0216385. doi: 10.1371/journal.pone.0216385 (PMC6497312; doi:10.1371/journal.pone.0216385)
Supplement: S3 File — Supporting data contain supplementary informations concerning the experiments on isolated rat heart ischemia and reperfusion. Raw data presents contractile activity (RPP), whole heart oxygen consumption (MVO2) during the pre-schemic and post-ischemic (reperfusion) phases for all the experiments, as well as all data used for the determination of infarct size. Separate files describe the results of all the statistical analyses presented in Figs 5 and 6. Finally, supplementary figures present pre-ischemic RPP and MVO2 and reperfusion phases (MVO2 and RPP to MVO2 ratio), as well as a graphic description of the protocols used in the study. (ZIP) [file pone.0216385.s003.zip › Heart perfusion (S3)/Heart Perfusion supplemental legend to figures.docx]

***Supplementary Heart Perfusion legend to figures.***

**Panel A:** Scheme summarizing the perfusion protocol used. Hearts were Langendorff-perfused, after the stabilization period hearts were either perfused with the vehicle (yellow box, control group) or with 10 µmol / L of OP2113 (green box, OP2113 group). Following 30 minutes of global normothermic ischemia (black box) and 120 minutes of reperfusion (gray box) hearts were stained to assess infarct size (red arrows). **Panels B** and **C:** These figures show the time course of MVO_2_ (panel B) and RPP to MVO_2_ ratio during the first 60 minutes of the reperfusion phase for the Control (black line) and the OP2113 (grey line) groups (n=6 in each group). **Panels D** and **E:** These figures show the time course of RPP (panel D) and MVO_2_ (panel E) during the pre-ischemic phase (stabilization + OP2113 or vehicle perfusion) for the Control (black line) and the OP2113 (grey line) groups (n=6 in each group).
