# Supplementary material for: An old medicine as a new drug to prevent mitochondrial complex I from producing oxygen radicals
Source: PLoS One. 2019 May 2;14(5):e0216385. doi: 10.1371/journal.pone.0216385 (PMC6497312; doi:10.1371/journal.pone.0216385)
Supplement: S3 File — Supporting data contain supplementary informations concerning the experiments on isolated rat heart ischemia and reperfusion. Raw data presents contractile activity (RPP), whole heart oxygen consumption (MVO2) during the pre-schemic and post-ischemic (reperfusion) phases for all the experiments, as well as all data used for the determination of infarct size. Separate files describe the results of all the statistical analyses presented in Figs 5 and 6. Finally, supplementary figures present pre-ischemic RPP and MVO2 and reperfusion phases (MVO2 and RPP to MVO2 ratio), as well as a graphic description of the protocols used in the study. (ZIP) [file pone.0216385.s003.zip › Heart perfusion (S3)/Heart Perfusion_Infarct size Methods.docx]

***Heart Perfusion***

All procedures conformed to the UK Animals (Scientific Procedures) Act 1986 and the Guide for the Care and Use of Laboratory Animals published by the National Institutes of Health (NIH Publication No. 85-23. revised 1996). Male Wistar rats (250-300 g) were anesthetized by 3% isoflurane, heparinized and euthanized by a lethal IP injection of pentobarbital (130 mg / kg) and hearts (~0.95 g of fresh wieght) were rapidly removed into ice-cold Krebs-Henseleit buffer containing (mmol / L): NaCl 118, NaHCO_3_ 25, KCl 4.8, KH_2_PO_4_ 1.2, MgSO_4_ 1.2, glucose 11 and CaCl_2_ 1.8, gassed with 95% O_2_ / 5% CO_2_ at 37°C (pH 7.4). Langendorff heart perfusions were performed as described previously (Garlid KD *et al.* Am J Physiol Heart Circ Physiol. 2006 Jul;291(1):H152-60). Hearts were perfused in a constant flow mode (12 ml / min) according to the protocol schematically described below. Global normothermic ischaemia (index ischaemia) was induced by halting perfusion for 30 min and immersing the heart in perfusion buffer at 37°C. At the end of the reperfusion period hearts were stained to assess infarct size, or freeze-clamped using liquid-nitrogen cooled tongues. In the latter case, hearts were grounded under liquid nitrogen, and stored at -80^o^C for later analysis.

***Assessment of infarct size***

At the end of the reperfusion period hearts were stained with triphenyltetrazolium chloride (TTC). Briefly, hearts were perfused for 7 min at 13 ml / min with a 12% (w / v) TTC solution to get 1% in the heart. Hearts were then detached from the cannula and incubated for an additional 4 min at 37°C before being sliced perpendicular to the longitudinal axis into 6 slices. The slices were then fixed in 4% (w / v) formalin solution overnight at 4°C and weighed. Both sides of each slice were photographed. The surface of the necrotic and area at risk of each side for each slice were determined by planimetry (AlphaEase v5.5), and since global ischemia was employed, infarct size was expressed as a percentage of the total cross-sectional area of the heart.

**Statistical analysis**

Data are expressed as means±SEM and represent 6 independent preparations. The n number in each group being less than 20 the distribution was considered non-normal and consequently a non-parametric Mann-Whitney test (SPSS statistics 17.0) was performed to compare the two groups. Results were considered statistically significant if the *p*-value was less than 0.05.
