# Supplementary material for: An old medicine as a new drug to prevent mitochondrial complex I from producing oxygen radicals
Source: PLoS One. 2019 May 2;14(5):e0216385. doi: 10.1371/journal.pone.0216385 (PMC6497312; doi:10.1371/journal.pone.0216385)
Supplement: S3 File — Supporting data contain supplementary informations concerning the experiments on isolated rat heart ischemia and reperfusion. Raw data presents contractile activity (RPP), whole heart oxygen consumption (MVO2) during the pre-schemic and post-ischemic (reperfusion) phases for all the experiments, as well as all data used for the determination of infarct size. Separate files describe the results of all the statistical analyses presented in Figs 5 and 6. Finally, supplementary figures present pre-ischemic RPP and MVO2 and reperfusion phases (MVO2 and RPP to MVO2 ratio), as well as a graphic description of the protocols used in the study. (ZIP) [file pone.0216385.s003.zip › Heart perfusion (S3)/Statistics/RPP_20min_Cont-Vehicle_VS_AOL.doc]

NPAR TESTS   /M-W= DataRPPContVehicle_AOL_20min BY RPPContVehvivle_AOL_20min(3 4)   /STATISTICS=DESCRIPTIVES   /MISSING ANALYSIS.


NPar Tests


Notes	
	Output Created	15-mars-2019 14:31:10	
	Comments		
Input	Data	C:\Documents and Settings\Administrateur\Bureau\Statistics_AOL_project.sav	
	Active Dataset	DataSet1	
	Filter	<none>	
	Weight	<none>	
	Split File	<none>	
	N of Rows in Working Data File	12	
Missing Value Handling	Definition of Missing	User-defined missing values are treated as missing.	
	Cases Used	Statistics for each test are based on all cases with valid data for the variable(s) used in that test.	
	Syntax	NPAR TESTS
  /M-W= DataRPPContVehicle_AOL_20min BY RPPContVehvivle_AOL_20min(3 4)
  /STATISTICS=DESCRIPTIVES
  /MISSING ANALYSIS.
	
Resources	Processor Time	0:00:00.000	
	Elapsed Time	0:00:00.000	
	Number of Cases Alloweda	112347	
a. Based on availability of workspace memory.	


[DataSet1] C:\Documents and Settings\Administrateur\Bureau\Statistics_AOL_project.sav


Descriptive Statistics	
	N	Mean	Std. Deviation	Minimum	Maximum	
DataRPPContVehicle_AOL_20min	12	3,135.5375	3,734.94252	16.62	10,437.45	
RPPContVehvivle_AOL_20min	12	3.50	.522	3	4	


Mann-Whitney Test


Ranks	
	RPPContVehvivle_AOL_20min	N	Mean Rank	Sum of Ranks	
DataRPPContVehicle_AOL_20min	3	6	6,83	41,00	
	4	6	6,17	37,00	
	Total	12			


Test Statisticsb	
	DataRPPContVehicle_AOL_20min	
Mann-Whitney U	16,000	
Wilcoxon W	37,000	
Z	-,320	
Asymp. Sig. (2-tailed)	,749	
Exact Sig. [2*(1-tailed Sig.)]	,818a	
a. Not corrected for ties.	
b. Grouping Variable: RPPContVehvivle_AOL_20min	
